# Population resequencing reveals candidate genes associated with salinity adaptation of the Pacific oyster *Crassostrea gigas*

**DOI:** 10.1038/s41598-018-26953-w

**Published:** 2018-06-06

**Authors:** Zhicai She, Li Li, Jie Meng, Zhen Jia, Huayong Que, Guofan Zhang

**Affiliations:** 1 Key Laboratory of Experimental Marine Biology, Institute of Oceanology, Chinese Academy of Sciences, Qingdao, 266071 Shandong China; 2 Laboratory for Marine Fisheries and Aquaculture, Qingdao National Laboratory for Marine Science and Technology, Qingdao, 266071 Shandong China; 3 Guangxi Key Laboratory of Beibu Gulf Marine Biodiversity Conservation, Qinzhou University, Qinzhou, 535011 Guangxi China; 4 Laboratory for Marine Biology and Biotechnology, Qingdao National Laboratory for Marine Science and Technology, Qingdao, 266071 Shandong China; 5 National & Local Joint Engineering Laboratory of Ecological Mariculture, Qingdao, 266071 Shandong China

## Abstract

The Pacific oyster *Crassostrea gigas* is an important cultivated shellfish. As a euryhaline species, it has evolved adaptive mechanisms responding to the complex and changeable intertidal environment that it inhabits. To investigate the genetic basis of this salinity adaptation mechanism, we conducted a genome-wide association study using phenotypically differentiated populations (hyposalinity and hypersalinity adaptation populations, and control population), and confirmed our results using an independent population, high-resolution melting, and mRNA expression analysis. For the hyposalinity adaptation, we determined 24 genes, including *Cg_CLCN7* (*chloride channel protein 7*) and *Cg_AP1* (*apoptosis 1 inhibitor*), involved in the ion/water channel and transporter mechanisms, free amino acid and reactive oxygen species metabolism, immune responses, and chemical defence. Three SNPs located on these two genes were significantly differentiated between groups, as was *Cg_CLCN7*. For the hypersalinity adaptation, the biological process for positive regulating the developmental process was enriched. Enriched gene functions were focused on transcriptional regulation, signal transduction, and cell growth and differentiation, including *calmodulin* (*Cg_CaM*) and *ficolin-2* (*Cg_FCN2*). These genes and polymorphisms possibly play an important role in oyster hyposalinity and hypersalinity adaptation. They not only further our understanding of salinity adaptation mechanisms but also provide markers for highly adaptable oyster strains suitable for breeding.

## Introduction

The complex and changeable environment of the intertidal zone is easily influenced by rainfall and land conditions, and shows a larger variation in temperature and salinity than other shore zones^1^. Salinity is one of the most important environmental factors, and varies considerably in intertidal zones^2^. Of the many species inhabiting the intertidal zone, those belonging to the phylum Mollusca are the most common^3^. The Pacific oyster *Crassostrea gigas* is representative of these intertidal organisms. Although this species has evolved mechanisms as adaptations to the intertidal environment, it is unable to withstand the dramatic fluctuations in salinity caused by alternating rainfall and drought, and therefore suffers large-scale mortality, especially in summer^4^, bringing losses to economy.

The Pacific oyster is an important cultivated shellfish, and is distributed along the north and south coasts of China. It is cultured mainly in small farms, where it has the characteristics of low input cost and high yield, and has become the main income source of coastal fishermen. When large-scale oyster mortality occurs, coastal fishermen therefore suffer serious economic losses. This would be alleviated by improved clarity concerning the salinity adaptation mechanisms and improvements in the resistance of oysters to salinity changes. Considerable efforts have been made in this respect to date; scientists have found two relatively independent systems of adaptation to different levels of environmental salinity changes. In molluscs, resistance is based mainly on an impeded water-salt exchange with the external medium, due to mantle cavity hermetization. Salinity tolerance in molluscs is determined by cellular adaptation mechanisms^5^. Free amino acids^6^, RNA and protein synthesis^7^, and inorganic ions such as sodium and chlorine^8–10^ have proven to be important factors in salinity adaptation. Molecular studies into cellular osmoregulatory related genes have been conducted^11,12^. Whole transcriptome responses to low salinity stress have been examined for the Pacific oyster^13^, and the functional mechanism of salt stress effectors has been analysed^4^. However, despite these previous efforts, the molecular mechanisms involved in salinity adaptation remain unknown.

Association analysis is a powerful tool for the dissection of complex traits and identification of alleles that contribute to a target trait^14^. Genome-wide association studies (GWAS) are able to use genome-wide scanning to identify either the genetic variation or gene that is associated with complex phenotypic traits, or traits that are economically valuable^15^. The advantages of GWAS are its high throughput, information content, and efficiency, allowing tens of thousands or more single nucleotide polymorphisms (SNPs) to be analysed at one time. GWAS can be applied to both quantitative and qualitative trait studies; case–control design is usually adopted for the latter. It requires a full genome draft and a sufficient number of molecular markers^16,17^; with the completion of genome sequencing in pattern and non-pattern organisms, and decreases in sequencing costs, it has been widely used in human, animal, and plant studies^18–21^. However, GWAS results often yield a high false positive, so the method is generally used in analysing the few associated SNPs present in another larger population, as well as mRNA expression in associated genes^22,23^. RNA sequencing is used for gene expression analysis in the whole genome, and for determining the genes associated with the target trait^24^. Recently, the availability of an oyster reference genome sequence^1^ and the development of high-throughput SNP assays^25^ have made it possible to use GWAS to explore the genetic basis of oyster salinity adaptation.

Thus, in the present study, we used GWAS with high density SNPs, genotyped by whole-genome resequencing in different salt stress populations, to identify genes and natural allelic variations that contribute to salt stress differentiation in oysters.

## Results

### Phenotypic differentiation

**Figure 1:**
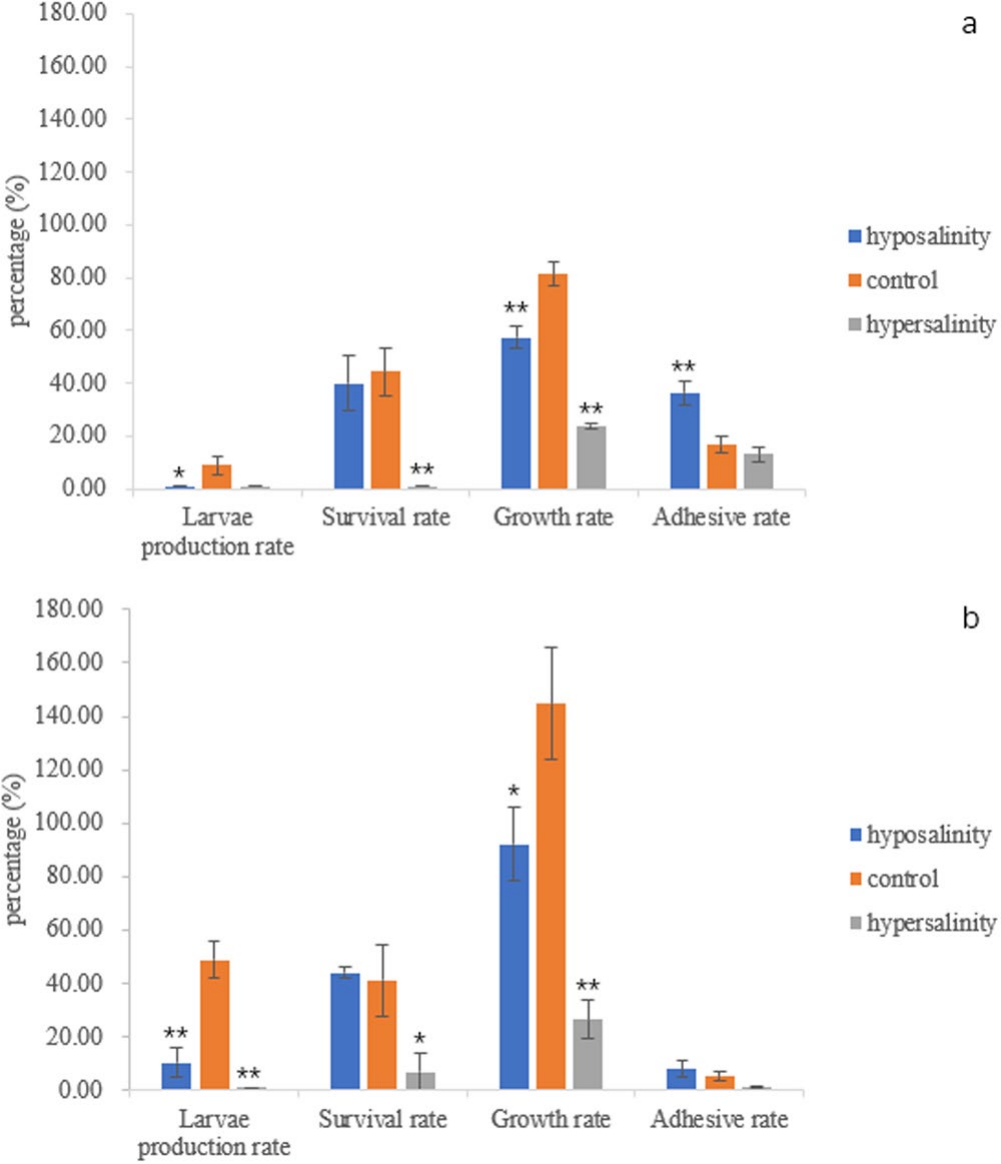
Comparative results of phenotypic traits between groups. (**a**) shows the phenotypic traits of the first set of experimental materials; (**b**) shows the phenotypic traits of the second set of experimental materials. “*” The difference reached a significant level, “**” The difference reached a highly significant level.

By constructing the experiments with animals and phenotypic differentiation assays at different larval stages, we obtained phenotypic data for different populations. Using a one-way ANOVA, we found that there was a significant difference between different populations (Fig. 1). In the first set of experimental materials, we found a significant difference for larvae production, growth, and adhesive rates between the hyposalinity adaptation and control population; the difference between the growth and adhesive rate reached a highly significant level. The survival and growth rates were also significantly different between the hypersalinity adaptation and control populations, both to a highly significant level. In the second set of experimental materials, the larvae production and growth rates in the hyposalinity adaptation population showed very significant differences compared to the control population; the same was also true for the hypersalinity adaptation population.

### Read alignment and SNP calling

DNA resequencing resulted in 462 million reads, or 93 Gb of raw sequencing data in total, 100% of which had quality scores at the CycleQ. 20 (a base quality greater than 20 and an error probability of 0.01) level. After filtering raw data based on high standards, we obtained high quality resequencing data. On average, about 76% of the reads covering 97% of the genome could be aligned to the genome; the depth ranged from 10 to 40×, mainly concentrated at about 35×. A total of 5.81, 5.94, and 5.64 million SNPs were identified in the hyposalinity adaptation, hypersalinity adaptation, and control populations, respectively.

### DNA differentiation for hyposalinity adaptation

For the hyposalinity adaptation, we obtained 681 differentiated SNPs and 934 differentiated genes, based on the allele frequency differentiation and *F*_ST_ analyses. We did not find any biological processes or KEGG (Kyoto Encyclopedia of Genes and Genomes) pathways related to hyposalinity adaptation, based on the enrichment analysis for the candidate gene set or expression profile. From the results of the GO (Gene Ontology Consortium; Supplementary Fig. S1) and COG (Clusters of Orthologous Groups; Supplementary Fig. S2) enrichment analyses, we found that differentiated genes were mainly concentrated on channel regulator and antioxidant activity, and signal transduction mechanisms.

In order to lock down the genes associated with hyposalinity adaptation, we drew a Venn diagram for three gene groups: the differentiated genes obtained by allele frequency differentiation and *F*_ST_ analysis, and the differentially expressed genes obtained by expression profile analysis under conditions of hyposalinity stress according to Meng *et al*.^4^. Finally, we screened a total of 157 differentiated genes using expression profiling under hyposalinity stress conditions (Supplementary Fig. S3). Among these 157 genes, we found several genes related to the ion/water channel and transporter mechanisms, free amino acid (FAA) and reactive oxygen species (ROS) metabolism, immunity responses, and chemical defence; we mainly focused on these genes (Table 1).

**Table 1:** Differentiated genes and single nucleotide polymorphisms associated with hyposalinity adaptation.

Gene classification	Gene ID	Gene annotation	SNPs	Genotype	Allele frequency difference	Scaffold	Position	Area
Ion/Water channel	OYG_10012229	Chloride channel protein 7	Cg_SNP_S1	Y	0.6074	scaffold190	78335	CDS
	OYG_10015111	Potassium voltage-gated channel protein	Cg_SNP_S2	W	0.6017	scaffold578	105942	Intergenic
	OYG_10024446	Aquaporin-2	Cg_SNP_S3	R	0.7609	scaffold784	833760	Intergenic
Transporter	OYG_10017584	Solute carrier family 22 member 13	Cg_SNP_S4	M	0.75	scaffold1402	278732	Intergenic
	OYG_10017841	Excitatory amino acid transporter 1	Cg_SNP_S5	W	0.6396	scaffold72	196168	Intron
	OYG_10028337	Taurine transporter	Cg_SNP_S6	Y	0.6428	scaffold419	251428	Intron
FAA metabolism	OYG_10011934	Ornithine aminotransferase	Cg_SNP_S7	K	0.6025	scaffold1720	23103	Intron
	OYG_10012828	Ornithine aminotransferase	Cg_SNP_S8	Y	0.6071	scaffold1603	315998	Intergenic
ROS-related	OYG_10004092	Extracellular superoxide dismutase [Cu-Zn]	Cg_SNP_S9	Y	0.8	scaffold1558	5417	Intergenic
	OYG_10004145	Lysozyme	Cg_SNP_S10	S	0.6666	scaffold40440	26183	Intron
	OYG_10007023	Glutathione peroxidase	Cg_SNP_S11	R	0.6795	scaffold42508	2001	Intergenic
Immune response	OYG_10005131	Baculoviral IAP repeat-containing protein 3	Cg_SNP_S12	R	0.6019	scaffold41522	22983	Intron
	OYG_10010262	Complement C1q-like protein 4	Cg_SNP_S13	R	0.6171	scaffold181	89888	Intron
	OYG_10011513	Apoptosis 1 inhibitor	Cg_SNP_S14	M	0.6667	scaffold43624	286034	Intron
	OYG_10013066	Complement C1q tumor necrosis factor-related protein 3	Cg_SNP_S15	K	0.6025	scaffold1000	4977	Intergenic
	OYG_10018846	Complement C1q-like protein 3	Cg_SNP_S16	Y	0.619	scaffold547	209775	Intron
	OYG_10019629	Scavenger receptor class F member 2	Cg_SNP_S17	Y	0.6421	scaffold1889	583732	Intron
	OYG_10027112	Apoptosis inhibitor IAP	Cg_SNP_S18	W	0.6111	scaffold433	1161722	Intron
Chemical defence	OYG_10003807	Heat shock 70 kDa	Cg_SNP_S19	Y	0.6071	scaffold40050	7148	Intergenic
	OYG_10004164	Stress-induced protein 1	Cg_SNP_S20	S	0.6333	scaffold1501	25676	Intron
	OYG_10011490	Cytochrome P450 1A2	Cg_SNP_S21	Y	0.6025	scaffold724	82719	Intron
	OYG_10014250	Cytochrome P450 3A9	Cg_SNP_S22	R	0.6307	scaffold1838	159533	Intergenic
	OYG_10022751	Heat shock 70 kDa protein 12 A	Cg_SNP_S23	S	0.6428	scaffold365	718925	Intron
	OYG_10023593	Cytochrome P450 2U1	Cg_SNP_S24	S	0.6019	scaffold20	650996	Intergenic

**Figure 2:**
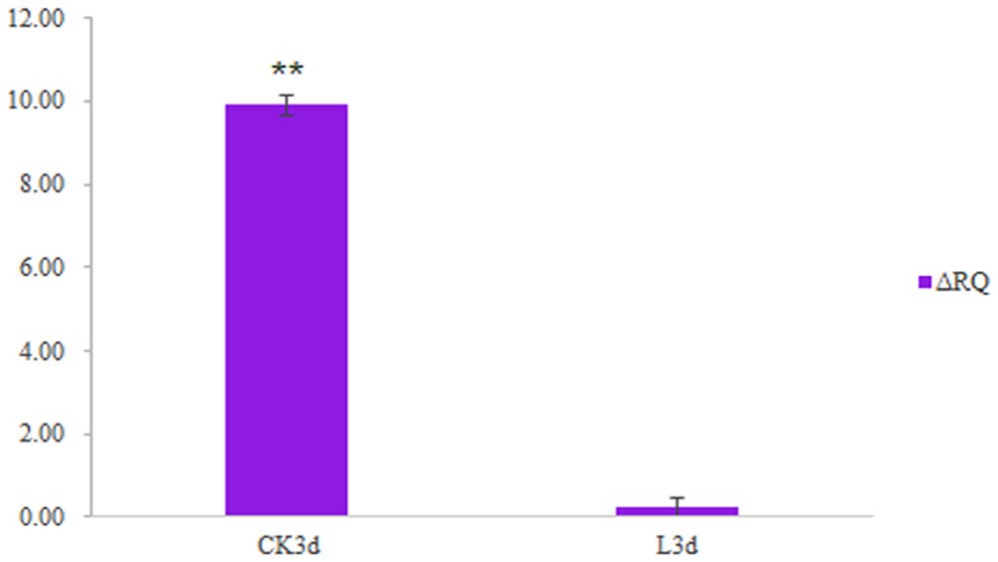
The expression variation in *Cg_CLCN7* between groups. CK3d, the expression variation in *Cg_CLCN7* of the control population on the third day; L3d, the expression variation in *Cg_CLCN7* of the hyposalinity adaptation population on the third day. “**” The difference reached a highly significant level.

**Figure 3:**
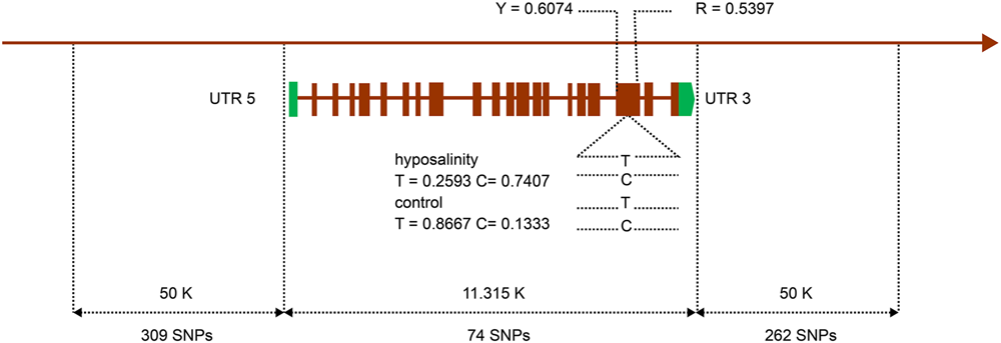
Distribution of single nucleotide polymorphisms on and around *Cg_CLCN7*: UTR 5, 5ʹ-untranslated regions; UTR 3, 3′-untranslated regions. Y, transition between C and T; R, transition between A and G.

Among the genes mentioned above, *Cg_CLCN7* contained an SNP locus Cg_SNP_S1, of which the allele frequency difference between the hyposalinity adaptation and control populations reached 0.6074; there was also another locus with an adjacent large allele frequency. In the populations used for validation, 40 SNPs (Supplementary Table S1) in total were successfully genotyped; one (Cg_SNP_SV34) was located downstream of *chloride channel protein 7 Cg_CLCN7*, 2231 bp from Cg_SNP_S1, of which the allele frequency difference reached 0.9317. Additionally, we undertook an expression analysis for *Cg_CLCN7* via qPCR, and found that the difference between populations reached a very significant level. Gene expression was upregulated by only 0.23 in the hyposalinity adaptation population, while in the control population it was upregulated by as much as 9.91 (Fig. 2). All of these results indicated that it was possible *Cg_CLCN7* and the SNPs Cg_SNP_S1 and Cg_SNP_SV34 play an important role in the process of hyposalinity adaptation in oysters. The distribution of SNPs with a large allele frequency difference on and around *Cg_CLCN7* is shown in Fig. 3.

In addition to *Cg_CLCN7*, the gene *Cg_AP1* also contained one SNP, Cg_SNP_S14, of which the allele frequency difference between the hyposalinity adaptation and control populations reached 0.6667; in the populations used for validation, the allele frequency difference reached 0.538. This indicated that Cg_SNP_S14 and the *Cg_AP1* on which it is located might also play an important role in the process of hyposalinity adaptation in oysters.

**Figure 4:**
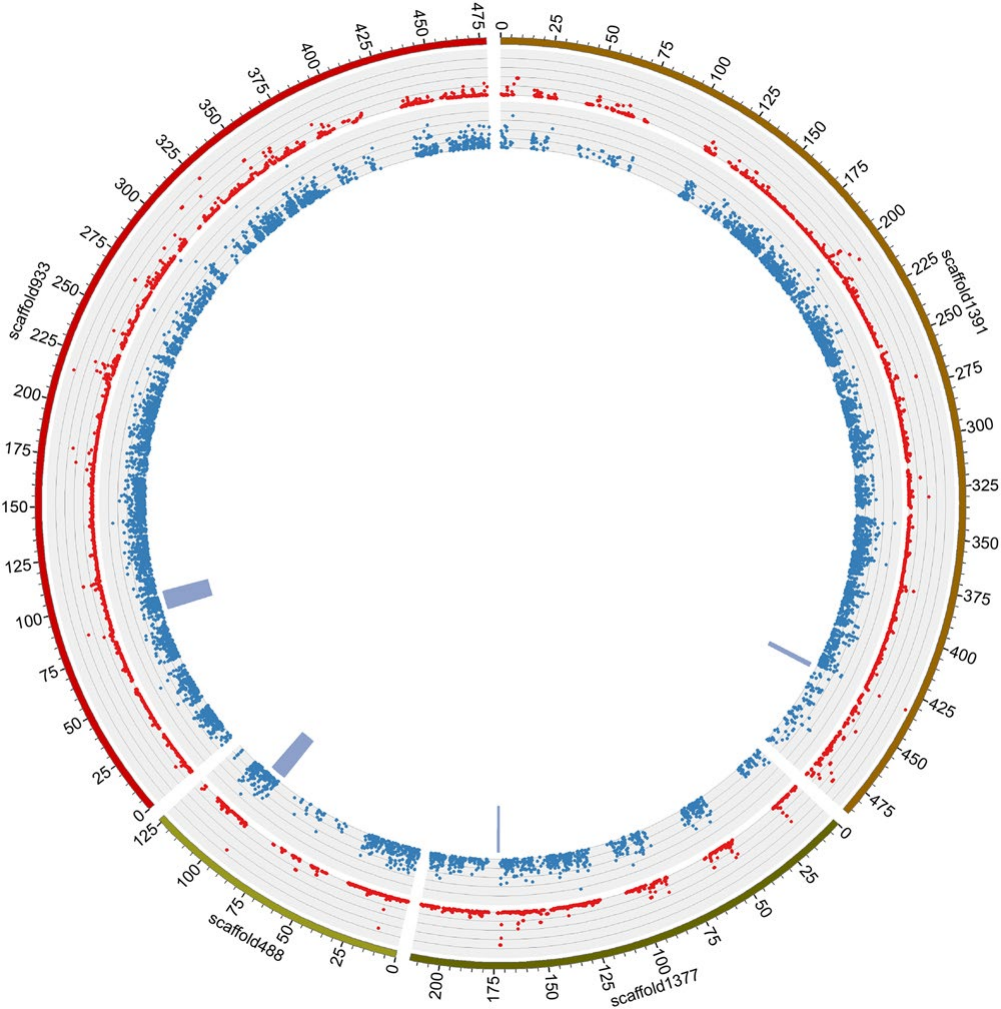
Circos analysis of important genes associated with hyposalinity adaptation: scaffold933, the scaffold that *Cg_FP1* located; scaffold488, the scaffold that *Cg_AIF1* located; scaffold1391, the scaffold that *Cg_CaM* located; scaffold1377, the scaffold that *protein IMPACT homolog* located. The first circle shows the length of the 4 scaffolds that the target genes located; the second circle shows the *F*_*ST*_ value; the third circle shows the allelic frequency difference of the differential SNPs; the fourth circle shows the location of target genes.

The *F*_ST_ analysis determined that calmodulin (*Cg_CaM*)*, protein IMPACT homolog, apoptosis-inducing factor 1* (*Cg_AIF1*), and *fibropellin-1* (*Cg_FP1*) all had very high *F*_ST_ and that there was high differentiation between populations. We selected these and used Circus software^26^ to show the SNP allele frequency difference and *F*_ST_ (Fig. 4).

### DNA differentiation for hypersalinity adaptation

**Figure 5:**
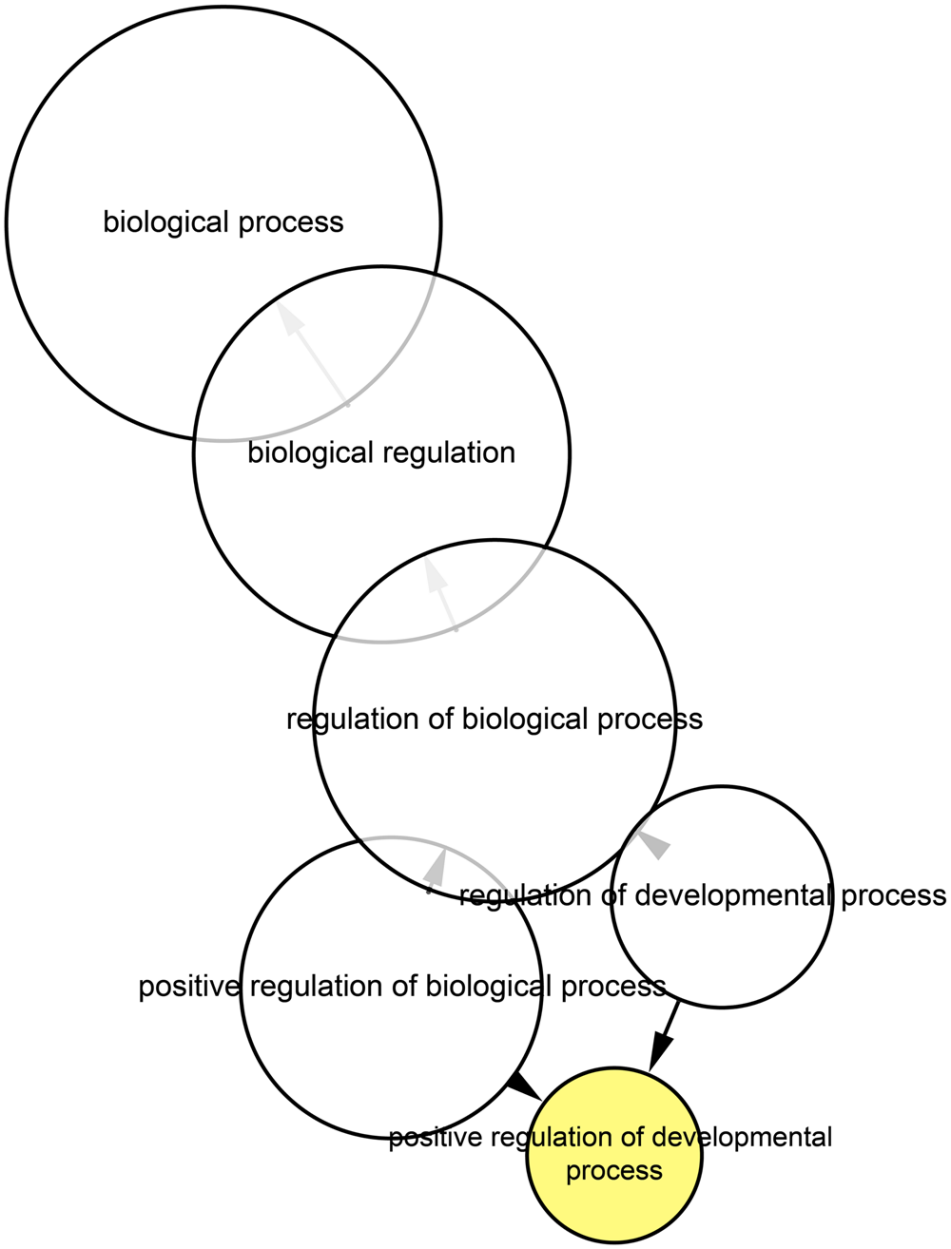
Enrichment analysis of the candidate differential gene set for the hypersalinity adaptation.

**Figure 6:**
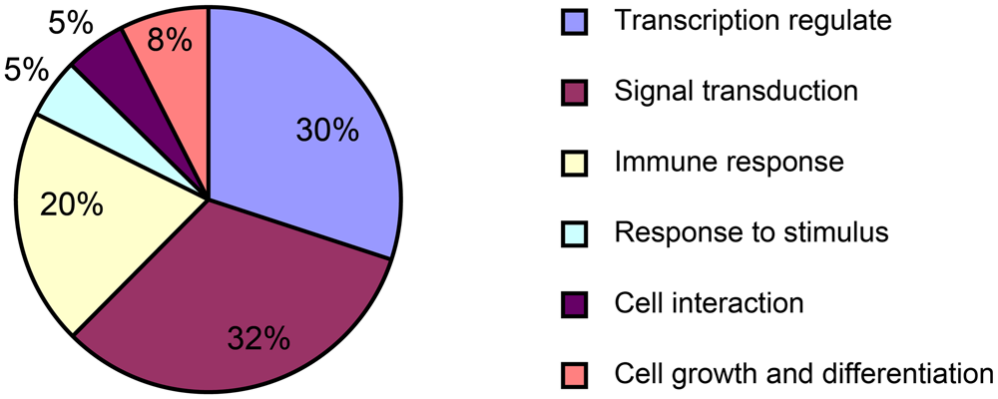
Functional classification of significantly enriched genes.

Based on the allele frequency differentiation analysis, we detected 438 differentiated SNPs located on 231 genes. Additionally, we found 558 differentiated genes, based on the *F*_ST_ analysis. Then, we merged the above two groups of genes into a single gene set containing 779 differentiated genes, on which we performed an enrichment analysis. As a result, we did not find any KEGG pathways related to hypersalinity adaptation, but we detected a significant difference in the positive regulation of the developmental process (Fig. 5), which indicated it might be related to hypersalinity adaptation. The functional classification of genes enriched in the biological process is shown in Fig. 6. Genes related to transcription regulation and signal transduction formed the highest proportion, while genes related to cell growth and differentiation accounted for eight percent.

**Figure 7:**
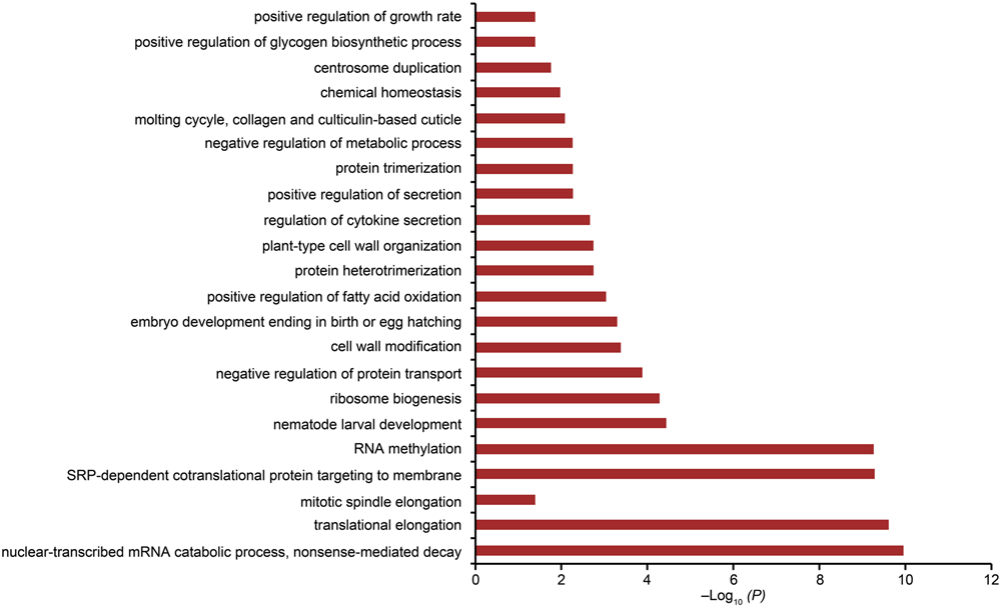
Distribution of P values for significantly enriched biological processes identified in the expression profiling of the hypersalinity group.

Based on the expression profile analysis, we detected 515 differentially expressed genes related to hypersalinity adaptation, and GO enrichment analysis showed that some biological processes were significantly enriched (P value shown in Fig. 7). Of these processes, positive regulation of growth rate and embryo development ending in birth or egg hatching were related to positive regulation of the developmental process, therefore supporting the finding that the latter might be related to hypersalinity adaptation at the expression level.

Based on *F*_ST_ analysis, *Cg_FCN2, Cg_CaM, Monocarboxylate transporter* (*Cg_MCT*), and *Cytochrome P450* (*Cg_CYP450*) revealed very high *F*_ST_ and were highly differentiated between populations. We selected these and showed the SNP allele frequency difference and *F*_ST_ using Circos (Supplementary Fig. S4).

## Discussion

The mechanisms underlying the adaptation of populations to their current environment is central to predicting their potential adaptive responses to environmental changes^27^. Comprehending these mechanisms is therefore helpful in improving the resistance of populations to such change. We conducted a genome-wide association study using phenotypic differentiation populations (hyposalinity adaptation population, hypersalinity adaptation population, and control population) to search for genes and their variants involved in the salinity adaptation of the Pacific oyster.

The major phenotypic differences between the hyposalinity adaptation, hypersalinity adaptation and control populations in the test group were basically consistent with those in the validation group, though there are certain differences between the two groups. There are many uncertainties that could influence the process of oyster development, and it’s really hard to achieve the same results of two independent artificial reproduction, maybe this is the reason that caused low repeatability of the treatment.

For the hyposalinity adaptation, we obtained the gene set that was potentially associated with responding to hyposalinity. There were 24 genes in total, and the related functions could be divided into the following six categories: ion/water channel and transporter mechanisms, FAA and ROS metabolism, immune response, and chemical defences. This is similar to previous studies, as follows. According to the research results of Meng *et al*.^4^, salt stress effectors mainly include ion channels, aquaporins, and FAA metabolism. Zhao *et al*. focus on biological functions such as osmoregulation, immune response, cell adhesion and communication, the cytoskeleton, cell cycle, and Ca^2+^-binding proteins^13^. In our study, we did not find any biological processes or KEGG pathways related to hyposalinity adaptation, based on the enrichment analysis for the candidate gene set or expression profile; this may be because the strength of the artificial stimulation was insufficient^5^. According to our data, the SNPs Cg_SNP_S1 and Cg_SNP_SV34 showed considerable differentiation between the hyposalinity adaptation and control populations, and might therefore play an important role in hyposalinity adaptation in oysters. The *Cg_CLCN7* gene on which they are located is also very important to hyposalinity adaptation, a role supported by its significant differential expression between groups (P < 0.01). Cl^−^, which is transported by *Cg_CLCN*, is one of the important ions involved in osmoregulation in oysters. Previous studies have shown that oysters regulate their intracellular concentration of solutes in order to adapt to surrounding conditions^5,28^, and that, under osmotic stress conditions, ion channels mediate an ionic steady state for Na^+^, Cl^−^, K^+^, and Ca^2+^^29^. *Cg_AP1*, which contains SNP Cg_SNP_S14, is also associated with hyposalinity adaptation. The allele frequency of Cg_SNP_S14 was differentiated in both the populations used for resequencing and those used for validation; this improved the reliability of the inference that *Cg_AP1* was differentiated during hyposalinity adaptation in oysters. *Cg_AP1* plays an important role in the immune response, which is in turn an important aspect of salinity stress responses in organisms^4,13^.

For the hypersalinity adaptation, we performed an enrichment analysis on the candidate gene set, and determined that the biological process related to positive regulation of development was enriched. Gene functions enriched in this process were focused on transcriptional regulation and signal transduction, which includes cell interaction, growth, and differentiation. Transcriptional regulation and signal transduction are found widely in salinity stress responses in organisms^30,31^. Through the expression profile analysis, we also determined biological processes that were related to larval growth and development, such as nematode larval development, embryo development ending in birth or egg hatching, and positive regulation of growth rate. We found enrichment of the positive regulation of developmental processes both at the DNA and RNA level; this might be because artificial stimulation was carried out during the eye-spot larval stage, which is very important to oyster development. The stimulation therefore had a significant effect on the development process, resulting in the related genes making corresponding regulations to help organisms adapt to adverse environments.

The genetic differentiations exist in the oyster population originally, and the selection makes the allele frequency and genotype frequency of the population change. Individuals with different genetic backgrounds have different adaptabilities to salinity stress; the artificial salinity stress gathered the similar ones together, as well as their similar genetic backgrounds, thus the genetic differentiations between different populations could be shown. Overall, phenotypic and genetic differentiations suggest that different populations may have different adaptive potential to environmental variations. The identification of these genes and polymorphisms not only furthers our understanding of salinity adaptation mechanisms in oysters, but also provides markers of highly adaptable oyster strains suitable for breeding, which would promote research related to oyster breeding, reduce economic losses, and increase the benefits of oyster culture.

## Materials and Methods

### Experimental animals and artificial stimulation

There were two sets of experimental materials in the whole experiment. The first was used to perform the association analysis and screen genges associated with salinity adaptation; the second was used to conform the results. The first set of materials was composed of 100 full-sib families crossed by 10 male and 10 female oysters, collected from Jiaonan, Qingdao, China. After fertilization, they were incubated in the same natural environmental conditions. After reaching the eye-spot stage, the larvae were divided into three equally sized groups, and cultured under 15 ppt, 45 ppt, and 30 ppt salinity, respectively. The larvae were fed four times a day and water was changed twice a day during the period of artificial stimulation. After eight days, all the groups were rehabilitated to a natural salinity level (30 ppt) and cultured in the same natural environmental conditions. Seven months later, we sampled 46 individuals from each group to do the DNA and RNA assay, these being the hyposalinity adaptation, hypersalinity adaptation, and control populations.

Using the same method as mentioned above, we produced a second set of experimental hyposalinity and hypersalinity adaptation populations and a control population, which contains three replicates and 300 full-sib families in total, and 90 individuals were sampled from each group to do the DNA and RNA assay. All the materials used in the whole experiment were shown in Table 2.

**Table 2:** Experimental materials used in the whole experiment.

	Salinity adaptation populations	Groups	Families	Phenotypic assay	DNA assay	RNA assay	Samples
the first set of experimental materials	hyposalinity	1	100	larval production, survival, growth, and adhesive rates	DNA resequencing	expression profile analysis	46
	control	1	100	larval production, survival, growth, and adhesive rates	DNA resequencing	expression profile analysis	46
	hypersalinity	1	100	larval production, survival, growth, and adhesive rates	DNA resequencing	expression profile analysis	46
the second set of experimental materials	hyposalinity	3	300	larval production, survival, growth, and adhesive rates	HRM	qPCR	180
	control	3	300	larval production, survival, growth, and adhesive rates	HRM	qPCR	270
	hypersalinity	3	300	larval production, survival, growth, and adhesive rates	HRM	qPCR	180

### Salinity adaptation differentiation assay

We tested four indexes to assay the salt resistance differentiation between the test and control populations, including larval production, survival, growth, and adhesive rates. Both the two sets of experimental materials were used to test the differentiation:

### Larvae production rate differentiation assay

After fertilization, eggs were removed from each group and divided equally into three subgroups for incubation at different salinities: 15 ppt, 30 ppt, and 45 ppt. After 24 hours, the number of D-stage larvae in each subgroup was recorded, and the larvae production rate was calculated. The other fertilized eggs were incubated under natural conditions until D-stage larvae were produced.

### Survival rate differentiation assay

After incubation, D-stage larvae were removed from each group and divided equally into three subgroups, and then cultured in 15 ppt, 30 ppt, and 45 ppt salinity conditions, respectively. After 18 days of this treatment, the number of surviving larvae in each subgroup was recorded, and the survival rate was calculated. The remaining D-stage larvae were cultured to the eye-spot stage under natural conditions.

### Growth rate differentiation assay

Before the D-stage larvae were divided, we selected 30 randomly from each group and measured shell height. After the treatment, we again measured the height of 30 shells randomly selected from each subgroup and the growth rate was calculated.

### Adhesive rate differentiation assay

Eye-spot larvae were removed from each group and equally divided into three subgroups, and then cultured in 15 ppt, 30 ppt, and 45 ppt salinity conditions, respectively. After eight days of this treatment, we recorded the number of adherent larvae in each subgroup and calculated the adhesive rate.

After obtaining the phenotypic data, we performed a one-way ANOVA to compare the phenotypic traits between different salinity adaptation populations.

### Whole genome resequencing

We extracted DNA using the E.Z.N.A SQ tissue DNA Kit (Omega Bio-tek, Inc., Norcross, GA, USA) from the mantle of the three populations: the hyposalinity and hypersalinity adaptation populations, and the control population. The DNA integrity was assessed by agarose gel electrophoresis; the DNA concentration was assessed using a spectrophotometer (Nanodrop 2000; Thermo Fisher Scientific, Wilmington, MA, USA). We selected 46 samples randomly for pooling from each population. A 101-bp paired-end sequencing library with a 300-bp insert size was constructed for each DNA pool by the Beijing Biomarker Technologies Corporation, according to standard Illumina protocols, and each DNA library was sequenced with the HiSeq. 2000 genome analyzer (Illumina, Inc., San Diego, CA, USA).

### Read alignment and SNP calling

The raw data was filtered to a high standard after evaluation, and were then aligned to the oyster genome with the Burrows-Wheeler Alignment Tool (BWA) software (http://bio-bwa.sourceforge.net/bwa.shtml)^32^. SNP calling was performed using SAMtools (http://samtools.sourceforge.net/samtools.shtml)^33^, according to the alignment results of the sequencing data and oyster genome. The filtering criteria was: a sequencing depth of no lower than two, and three for heterozygous SNP; a maximum depth of no more than three times the average depth; a base quality higher than 20; and located outside the simple sequence repeat region.

### SNP allele frequency differentiation analysis

We calculated the allele frequency for each SNP locus, and determined the difference in allele frequency between different populations. Then, we extracted SNPs that showed different allele frequencies during pairwise comparison in both the hypo- and the hypersalinity adaptation populations. SNPs with a difference above 0.6 were selected as differentiated SNPs for salinity adaptation, and the genes on which these SNPs were located were selected as differentiated genes.

### *F*_ST_ analysis

We also calculated *F*_ST_ for all the 1000-bp windows on the genome. Then, the windows were sorted from high to low according to *F*_ST_, and those in the highest 1% were selected. We then extracted genes that overlapped with these selected windows, together with 5 Kb of both upstream and downstream of the windows, for both the hypo- and the hypersalinity adaptations.

### Gene set enrichment analysis

Genes obtained from the SNP allele frequency differentiation and *F*_ST_ analyses were merged into a gene set for the hypo- and the hypersalinity adaptation, respectively; these were then used to undertake GO, COG, and the KEGG enrichment analyses. From the enrichment analysis, we determined the candidate gene set for hypo- and hypersalinity adaptation.

### Expression profile analysis

Invitrogen TRIzol reagent (Thermo Fisher Scientific, Wilmington, MA, USA) was used to extract the total RNA from the gills of the oysters used for whole genome resequencing. For each of the three populations, the total RNA of all 46 individuals was mixed together in equal amounts, and used to construct a sequencing library, which was then sequenced using the HiSeq. 2000 genome analyzer (Illumina, Inc., San Diego, CA, USA). First, the sequencing data was aligned to the reference gene sequence after evaluation using Blast software (The BLAST-Like Alignment Tool, http://genome.ucsc.edu/cgi-bin/hgBlat)^34^, and we carried out a quantitative analysis of gene expression according to the alignment results. Second, we used EBSeq software^35^ to detect differentially expressed genes according to the quantitative results. Third, we compared the sequences of the differentially expressed genes with NR (non-redundant), Swiss-Prot, GO, COG, and KEGG databases, using BLAST software for gene annotation. Finally, a COG, GO, and KEGG enrichment analysis was performed for the differentially expressed genes.

### Validation of the candidate polymorphisms and genes

We constructed 300 full-sib families crossed by 30 male and 30 female individuals collected from Jiaonan, Qingdao, China, and performed artificial stimulation to obtain another three salinity adaptation differentiated populations: hyposalinity and hypersalinity adaptation populations, and the control population, as well as the salinity adaptation differentiation assay. This was undertaken in the same way as previously described.

Differentiated SNPs (Table 1), and those developed in the partial differentiated genes (Supplementary Table S2) that were obtained from the SNP allele frequency differentiation analysis, were selected for validation. We used high-resolution melting (HRM) analysis^36^ for SNP development and genotyping in the three salinity adaptation differentiated populations, and then carried out SNP allele frequency differentiation analysis to determine whether it was the same as in the populations used for resequencing.

The differentiated genes (Supplementary Table S2) were also validated at the RNA level. We carried out artificial stimulation on the three populations: hyposalinity stimulation (15 ppt) on the hyposalinity adaptation and control populations, and hypersalinity stimulation (45 ppt) on the hypersalinity adaptation and control populations. For hyposalinity stimulation, we sampled 90 individuals from each population at zero hours as a control group, and then sampled another 90 individuals after three days as the test group, from each treatment. For hypersalinity stimulation, we used the same method as for the hyposalinity stimulation. Then, a quantitative polymerase chain reaction (qPCR) was performed to assay the expression difference of the differentiated genes between the control and test groups.

### Data availability

The oyster genome is available at http://www.oysterdb.com/FrontHomeAction.do?method=home.

## Electronic supplementary material

Supplementary Information
