# Supplementary material for: Population resequencing reveals candidate genes associated with salinity adaptation of the Pacific oyster Crassostrea gigas
Source: Sci Rep. 2018 Jun 6;8:8683. doi: 10.1038/s41598-018-26953-w (PMC5989259; doi:10.1038/s41598-018-26953-w)
Supplement: Supplementary file 1 — Supplementary Information [file 41598_2018_26953_MOESM1_ESM.pdf]

Population resequencing reveals candidate genes associated with salinity adaptation of the Pacific oyster *Crassostrea gigas*

Zhicai She[3], Li Li[1, 2, 5, *], Jie Meng[1, 2, 5], Zhen Jia[3], Huayong Que[1, 5], Guofan Zhang[1, 4, 5, *]

[1]*Key Laboratory of Experimental Marine Biology, Institute of Oceanology, Chinese Academy of Sciences, Qingdao 266071, Shandong, China*

[2]*Laboratory for Marine Fisheries and Aquaculture, Qingdao National Laboratory for Marine Science and Technology, Qingdao 266071, Shandong, China*

[3]*Guangxi Key Laboratory of Beibu Gulf Marine Biodiversity Conservation, Qinzhou University, Qinzhou 535011, Guangxi, China*

[4]*Laboratory for Marine Biology and Biotechnology, Qingdao National Laboratory for Marine Science and Technology, Qingdao 266071, Shandong, China*

[5]*National& Local Joint Engineering Laboratory of Ecological Mariculture, Qingdao 266071, Shandong, China*

*Correspondence to:

Dr. Li Li and Dr. Guofan Zhang

Institute of Oceanology, Chinese Academy of Sciences

7 Nanhai Rd., Qingdao 266071, China

Tel: +86-0532-82898701; Fax: +86-0532-82898701.

E-mail: lili@qdio.ac.cn (Li Li); gfzhang@qdio.ac.cn (Guofan Zhang)

Supplementary information

Supplementary Fig. S1. Results of the GO (Gene Ontology Consortium) enrichment analysis of differential genes in the hyposalinity group.

Supplementary Fig. S2. COG (Clusters of Orthologous Groups) function classification of differential genes in the hyposalinity group.

Supplementary Fig. S3. Venn diagram of the differential genes from resequencing and salinity stress expression profiling: AFD genes, differentiated genes obtained by allele frequency differentiation analysis; F_{ST} genes, differentiated genes obtained by F_{ST} analysis; DEG genes, differentiated genes obtained by expression profile analysis.

Supplementary Fig. S4. Circos analysis of the important genes associated with hypersalinity adaptation: scaffold77, the scaffold that *Cg_CYP450* located; scaffold42896, the scaffold that *Cg_ FCN2* located; scaffold1391, the scaffold that *Cg_CaM* located; scaffold544, the scaffold that *Cg_MCT* located. The first circle shows the length of the 4 scaffolds that the target genes located; the second circle shows the F_{ST} value; the third circle shows the allelic frequency difference of the differential SNPs; the fourth circle shows the location of target genes.

Supplementary Table S1. Information for the 40 genotyped single nucleotide polymorphisms in the populations used for confirmation of results.

Supplementary Table S2. List of candidate genes for validation at DNA and RNA level.

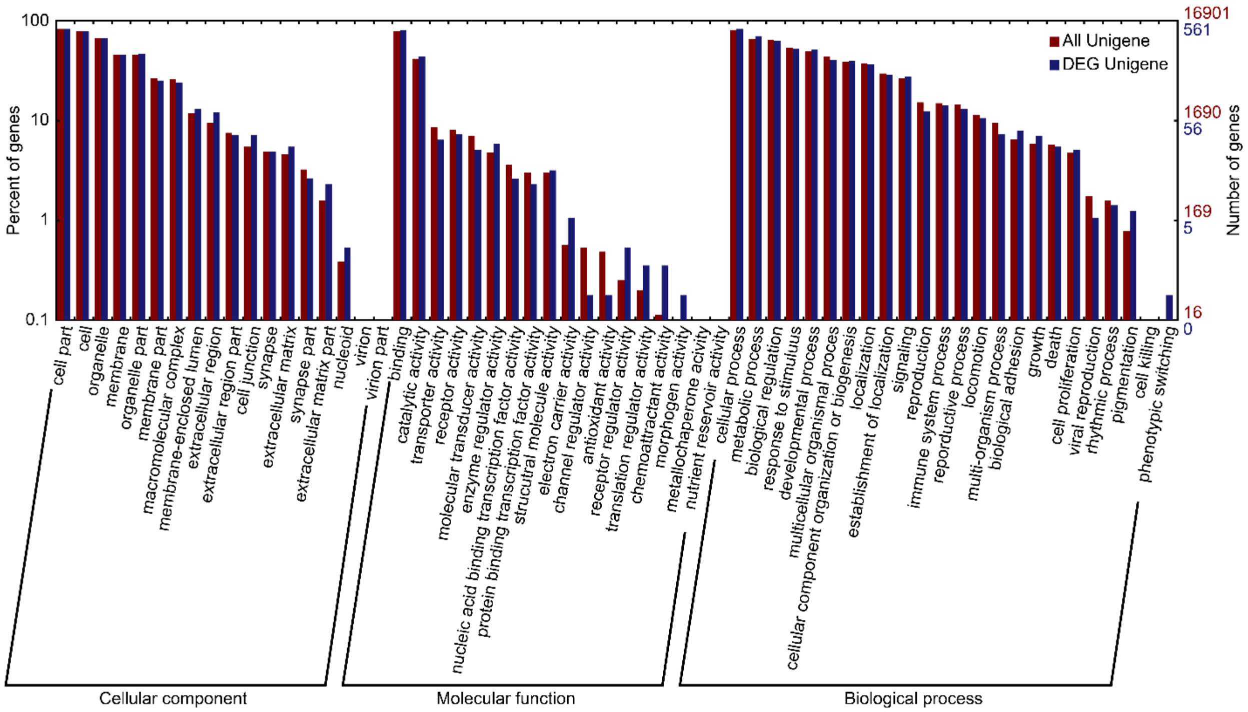

Supplementary Fig. S1 Results of the GO (Gene Ontology Consortium) enrichment analysis of differential genes in the hyposalinity group

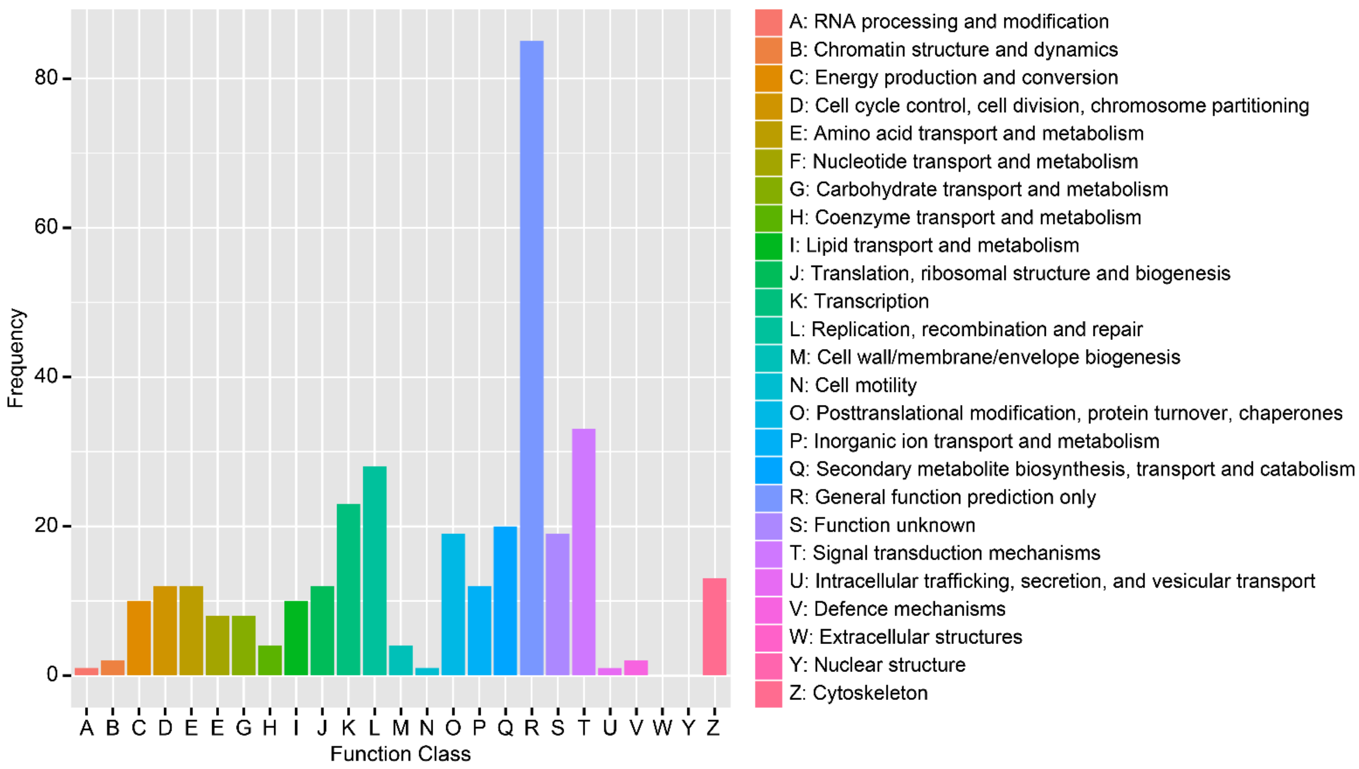

Supplementary Fig. S2 COG (Clusters of Orthologous Groups) function classification of differential genes in the hyposalinity group

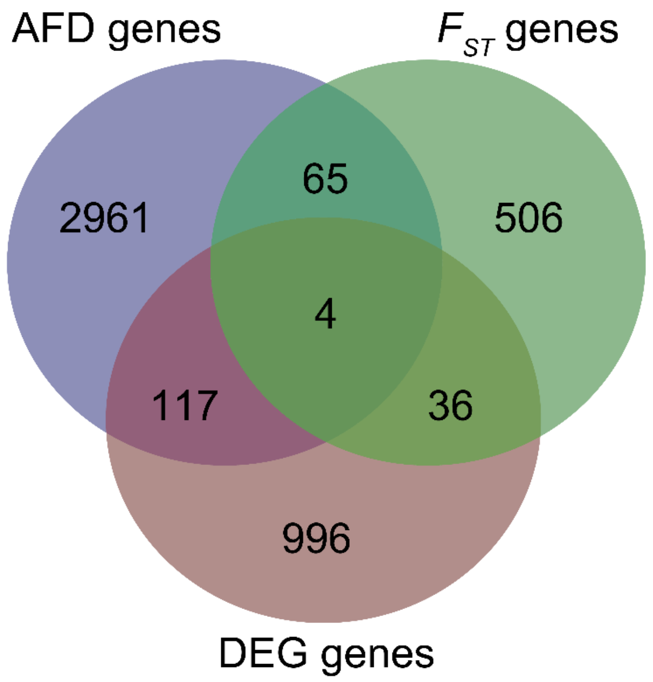

Supplementary Fig. S3 Venn diagram of the differential genes from resequencing and salinity stress expression profiling: AFD genes, differentiated genes obtained by allele frequency differentiation analysis; F_{ST} genes, differentiated genes obtained by F_{ST} analysis; DEG genes, differentiated genes obtained by expression profile analysis.

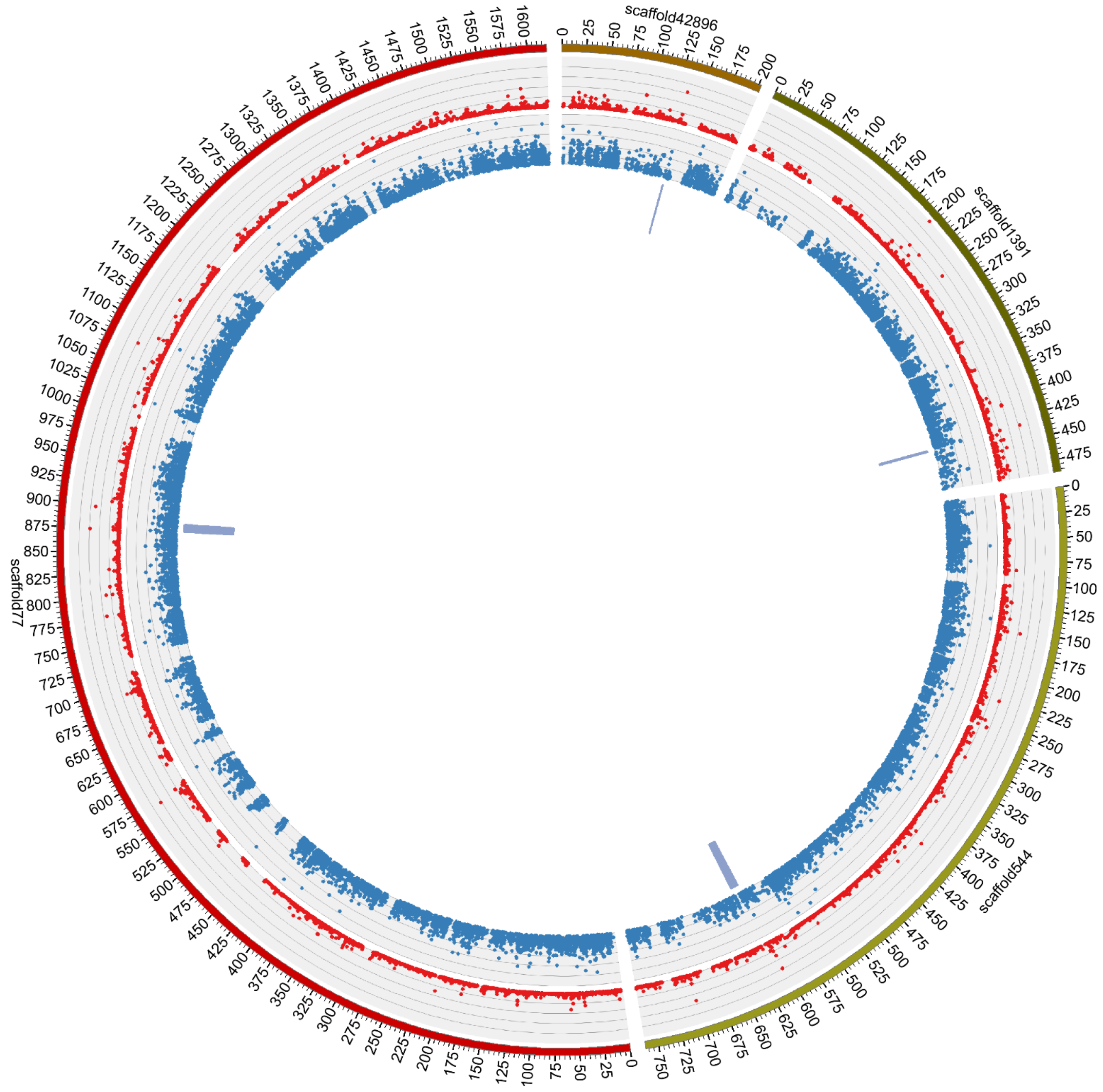

Supplementary Fig. S4 Circos analysis of the important genes associated with hypersalinity adaptation: scaffold77, the scaffold that *Cg_CYP450* located; scaffold42896, the scaffold that *Cg_ FCN2* located; scaffold1391, the scaffold that *Cg_CaM* located; scaffold544, the scaffold

that *Cg_MCT* located. The first circle shows the length of the 4 scaffolds that the target genes located; the second circle shows the F_{ST} value; the third circle shows the allelic frequency difference of the differential SNPs; the fourth circle shows the location of target genes.

Supplementary Table S1 Information for the 40 genotyped single nucleotide polymorphisms in the populations used for confirmation of results

SNP	Alleles	Gene_ID	Scaffold	Position	Forward primer(5′-3′)	Reverse primer(5′-3′)
Cg_SNP_SV2	G/T	CGI_10011934	scaffold1720	23103	GCTTTTGGATGAACGA	CGAAAATTGTATGGAT
Cg_SNP_SV5	A/C	CGI_10011513	scaffold43624	286034	GCTTGTTGATGCCTGA	AATTTATGCACAACTGACA
Cg_SNP_SV7	C/T	CGI_10019629	scaffold1889	583732	GTGTTATACAACCCGCTTAT	CGTATTATTGTAAAATGATTCC
Cg_SNP_SV13	C/T	CGI_10012229	scaffold190	74761	GGATAATTGCCTCCCTTC	AAGACATATGGTATGCCT
Cg_SNP_SV26	G/T	CGI_10012229	scaffold190	78232	GATGGTTTCTTTGACAAC	CATCTCAAATACATTCCA
Cg_SNP_SV34	G/T	CGI_10012229	scaffold190	80566	ATCAAGACTTGTACTTTG	TACCATCATGTGACATAG
Cg_SNP_SV36	C/T	CGI_10012229	scaffold190	81470	CAAGCATGTCTTAATTGT	CATGAAATATCTTATGCA
Cg_SNP_SV44	G/T	CGI_10012229	scaffold190	82270	GACAGTTAACAGTGATGTCCGA	TCGTTGACCGGAATGTAA
Cg_SNP_SV45	A/G	CGI_10012229	scaffold190	82379	AAGATGGAGAAAATTCGG	ATTGGTCCTGATGTAAAA
Cg_SNP_SV50	A/G	CGI_10015111	scaffold578	100987	TATTCAGTGATAAATGCTTC	TCAATATCTGATTCCCAA
Cg_SNP_SV51	G/T	CGI_10015111	scaffold578	101389	GAGGGGCCTATTTACCTG	CCATTCATAAACCAAGTC
Cg_SNP_SV53	C/T	CGI_10015111	scaffold578	101767	AGTTTATTTCATGGCTTA	GTTAAAGCATTAATTCAC
Cg_SNP_SV54	G/T	CGI_10015111	scaffold578	102220	ACTGGTGAATTATTAGATAC	CTGGAATCAGTAGACAAA
Cg_SNP_SV56	C/T	CGI_10015111	scaffold578	102310	GGACTATAGGCATAAATC	CTATGTCCTCATTCTTTT
Cg_SNP_SV57	C/T	CGI_10015111	scaffold578	103123	AAAATACCCGTGTAAATAG	GCAGCAGCAGAAAACCTC
Cg_SNP_SV66	A/G	CGI_10015111	scaffold578	104772	GCTTTGTTCCCTTGTCAG	TGCACGTATTCATAATACTGAG
Cg_SNP_SV69	C/T	CGI_10015111	scaffold578	106591	ACTCCCATTCCTCCTTTT	ATCATGTGACACTTTCTG
Cg_SNP_SV75	A/G	CGI_10015111	scaffold578	107074	AAGTTTCAACTGAGGAAG	GCACAGAGGCAATAATAG
Cg_SNP_SV76	A/G	CGI_10015111	scaffold578	107313	GGGATGTGGATGTTTAAC	CCTTAACTATGTACACGTAAC
Cg_SNP_SV86	G/T	CGI_10015111	scaffold578	108480	GGTCTATTACTGGCTTTG	AGTCCACTATTTCCCTATT
Cg_SNP_SV87	A/G	CGI_10015111	scaffold578	108844	GAATTGGGAATAAAGAAAAG	AGGTATTGGCATTGGTTG
Cg_SNP_SV92	A/G	CGI_10015111	scaffold578	109483	TGCTGCTTATAAAGACTC	AGATTAGGATGTTGATTG

Cg_SNP_SV100	C/T	CGI_10024446	scaffold784	829093	TCAAAACCAATCTACTCA	TAGGAATTCATTATCCAG
Cg_SNP_SV102	G/T	CGI_10024446	scaffold784	829277	TTGATGCAGTCTACATTGT	GCCGATTTAGATAGAACA
Cg_SNP_SV103	C/T	CGI_10024446	scaffold784	829566	ATGGGGATAATCTTCGGTA	GATATTGAAAACGTTAAAAC
Cg_SNP_SV104	A/C	CGI_10024446	scaffold784	829752	TTCTGCCTTCGGATACAC	TTCCTCGTTTGAAATTGG
Cg_SNP_SV108	C/T	CGI_10024446	scaffold784	833952	CACCTTATTTTGTCAAATCCTA	TGCCAAGCATGGACAGTT
Cg_SNP_SV133	A/C	CGI_10017841	scaffold72	193648	CCTCAGTGTAGTTCTCAAT	TTAGAGGATGTGAATGTATC
Cg_SNP_SV137	C/T	CGI_10017841	scaffold72	195049	GACCTACATTTATTTTCTGGCT	GGAGAAAGAACTGGAGGC
Cg_SNP_SV141	A/G	CGI_10017841	scaffold72	197148	CCCCTTATCAACCTTACC	TGAAACAGTACAGAATGCC
Cg_SNP_SV144	C/T	CGI_10017841	scaffold72	197513	CAGGGAAGATAACAGGAA	GAACAGGAGCTTGCGTAC
Cg_SNP_SV146	C/T	CGI_10017841	scaffold72	197875	ACTTTGGGTTGACCTTGA	GTGATGACTGCGGTGTTA
Cg_SNP_SV147	A/G	CGI_10017841	scaffold72	199223	AACTTACTTGTTGTCCTTCA	ATCCAGACTTCTATTCACTATG
Cg_SNP_SV150	C/T	CGI_10017841	scaffold72	201040	CTTTGCTTCCTCCTGTTG	CACTAAGTTTACTTTGTGGT
Cg_SNP_SV158	C/T	CGI_10028337	scaffold419	249201	CTTAGGCTAGGGATGCTT	AACCACTGGGTCAATTTC
Cg_SNP_SV159	A/G	CGI_10028337	scaffold419	249252	TTGAAATTGACCCAGTGG	TCCGTCTGTCTGTAAACTTTT
Cg_SNP_SV166	A/C	CGI_10028337	scaffold419	253053	CATAGTAGTCTGTATCTTGCCC	TACTGTCGGTCCGTCTGT
Cg_SNP_SV178	A/G	CGI_10028337	scaffold419	255426	CATTCGTCTCATTCCTTT	TGTTGGCTAACAGTGTTG
Cg_SNP_SV179	A/G	CGI_10028337	scaffold419	255459	GTAGTTCAACACTGTTAGCCAA	CCTGATGACCCCATTCTG
Cg_SNP_SV181	A/G	CGI_10028337	scaffold419	255542	TCGTCCAGGACCACTTTAGT	CTGAAGCGCCACCAGCTC

Supplementary Table S2 List of candidate genes for validation at DNA and RNA level

Gene ID	Annotation
CGI_10012229	Chloride channel protein 7
CGI_10015111	Potassium voltage-gated channel protein
CGI_10024446	Aquaporin-2
CGI_10017584	Solute carrier family 22 member 13
CGI_10017841	Excitatory amino acid transporter 1
CGI_10028337	Taurine transporter
CGI_10011513	Apoptosis 1 inhibitor
